# Inter-placental variability is not a major factor affecting the healing efficiency of amniotic membrane when used for treating chronic non-healing wounds

**DOI:** 10.1007/s10561-023-10096-y

**Published:** 2023-05-25

**Authors:** Vojtech Horvath, Alzbeta Svobodova, Joao Victor Cabral, Radovan Fiala, Jan Burkert, Petr Stadler, Jaroslav Lindner, Jan Bednar, Martina Zemlickova, Katerina Jirsova

**Affiliations:** 1 Department of Vascular Surgery, Na Homolce Hospital, Prague, Czech Republic; 2 2nd Department of Surgery – Department of Cardiovascular Surgery, First Faculty of Medicine, Charles University and General University Hospital in Prague, Prague, Czech Republic; 3 Laboratory of the Biology and Pathology of the Eye, Institute of Biology and Medical Genetics, First Faculty of Medicine, Charles University and General University Hospital in Prague, Albertov 4, 128 01 Prague, Czech Republic; 4 Department of Cardiovascular Surgery, Motol University Hospital, Prague, Czech Republic; 5 Department of Transplantation and Tissue Bank, Motol University Hospital, Prague, Czech Republic; 6 Clinic of Dermatovenerology, General Teaching Hospital and 1st Faculty of Medicine, Charles University, Prague, Czech Republic

**Keywords:** Placenta, Amniotic membrane, Wound healing efficiency

## Abstract

This study aimed to evaluate the efficacy of cryopreserved amniotic membrane (AM) grafts in chronic wound healing, including the mean percentage of wound closure per one AM application, and to determine whether the healing efficiency differs between AM grafts obtained from different placentas. A retrospective study analyzing inter-placental differences in healing capacity and mean wound closure after the application of 96 AM grafts prepared from nine placentas. Only the placentas from which the AM grafts were applied to patients suffering from long-lasting non-healing wounds successfully healed by AM treatment were included. The data from the rapidly progressing wound-closure phase (p-phase) were analyzed. The mean efficiency for each placenta, expressed as an average of wound area reduction (%) seven days after the AM application (baseline, 100%), was calculated from at least 10 applications. No statistical difference between the nine placentas’ efficiency was found in the progressive phase of wound healing. The 7-day average wound reduction in particular placentas varied from 5.70 to 20.99% (median from 1.07 to 17.75) of the baseline. The mean percentage of wound surface reduction of all analyzed defects one week after the application of cryopreserved AM graft was 12.17 ± 20.12% (average ± SD). No significant difference in healing capacity was observed between the nine placentas. The data suggest that if there are intra- and inter-placental differences in AM sheets’ healing efficacy, they are overridden by the actual health status of the subject or even the status of its individual wounds.

## Introduction

For years, the human amniotic membrane (AM) has become widely used as a bioactive dressing or the basic substrate for producing broadly distributed derivatives with beneficial healing properties (Fenelon et al. 2021; Nejad et al. 2021; Elkhenany et al. 2022). While AM transplantation was primarily adopted in ophthalmology for the reconstruction of the ocular surface (corneal ulcers, persistent epithelial defects, limbal stem cell deficiency, ocular neoplasia, pterygium), and for ocular surface wound healing (e.g., for chemical and thermal injuries, dry eye disease, recurrent corneal erosions or cicatrizing conjunctivitis such are Steven’s Johnson syndrome, toxic epidermal necrolysis, pemphigoid or graft versus host disease) (Tsubota et al. 1996; Fuchsluger et al. 2005; Meller et al. 2011; Tabatabaei et al. 2017; Walkden 2020). Later its application has been extended to the problem of healing wounds other than that of the eye, and its use has been developing strongly over the last few decades (DiDomenico et al. 2016; Johnson et al. 2021). The primary material for AM acquisition, the placenta, is readily available and relatively abundant compared to other transplants (Jirsova and Jones 2017). AM’s anti-inflammatory, anti-fibrotic, anti-microbial, neurotrophic, analgesic, anti-, and pro-angiogenic properties, along with the epithelization promotion, make it an ideal material for treating a wide variety of wounds (Wassmer and Berishvili 2020; Elkhenany et al. 2022). The rationale behind most of the mentioned effects has been characterized (Baradaran-Rafii et al. 2013), although the presence of substances, which can be responsible for the direct analgesic effect of AM, has only recently been suggested (Svobodova et al. 2023)**.** The AM immunogenicity is very low; thus, the risk of rejection or incompatibility complication is practically non-existent (Adinolfi et al. 1982; Hori et al. 2006).

The efficiency of AM is assigned to the presence of extracellular matrix proteins, a variety of growth factors, and cytokines, the production of which can direct adhesion, migration, proliferation, and differentiation of epithelial and stromal cells, as well as the stem and progenitor cells of epithelial and mesenchymal origin (Koizumi et al. 2000a, b; Bomfim Pereira et al. 2016; Wassmer and Berishvili 2020; Ruiz-Canada et al. 2021).

However, this also suggests that the properties of the AM prepared for transplantation will be dependent on many factors that can influence the production, concentration, and activity preservation of these substances in the AM. AM quality can be influenced by factors related to the donor/placenta-specific variations (Hopkinson et al. 2006a, b; Krabcova et al. 2014; Deihim et al. 2016) and by the handling dependent/induced factors (Allen et al. 2013; Paolin et al. 2016). The formers are responsible for both donor-dependent (inter-placental) variations (Hopkinson et al. 2006a, b; Krabcova et al. 2014) and intra-placental sub-region variations of AM composition (Deihim et al. 2016; Litwiniuk et al. 2018; Moraes et al. 2021). They can be influenced by the donor’s overall physiological status, genetic predisposition, the presence of pathology, or even by the week of pregnancy at which the placenta was retrieved (Skinner et al. 1981; Tossetta et al. 2014).

Studies evaluating the effect of intra- or inter-placental variations are relatively scarce. The evaluation of the properties of placental subregions was documented in several studies, describing the sub-regional differences from different aspects: the presence of stem cell markers (Lemke et al. 2017; Centurione et al. 2018; Garcia-Lopez et al. 2019), proliferation and differentiation capacity (Germain et al. 1992; Curtis et al. 1997; Farrugia et al. 2000; Han et al. 2008; Kim et al. 2011; Centurione et al. 2018), factors influencing wound healing and angiogenesis (Han et al. 2008; Gicquel et al. 2009; Lee et al. 2010; Banerjee et al. 2018; Litwiniuk et al. 2018) and other factors.

It was suggested that AM from placental and reflected sub-regions might have different potentials for tissue regeneration due to the different mitochondrial activity, which may be, in turn, crucial for clinical applications (Banerjee et al. 2015). Similarly, the study based on the evaluation of TGF*β*s (1,2,3) presence discovered significant differences in their concentration among individual donors proposing a potential modification of the healing effect based on the donor individuality (Hopkinson et al. 2006a, b; Han et al. 2008). Another study evaluated the placenta quality dependent on the pregnancy week retrieval (Skinner et al. 1981). Contrary, no significant difference in pluripotency markers concentration was found between the placental and reflected amnion (Garcia-Lopez et al. 2019), suggesting the homogeneous distribution of the pluripotency transcription factors, making all regions of AM equal in the regenerative processes effect. For an excellent review analyzing the data concerning the AM sub-regional differences, see Weidinger et al. (2020).

The studies evaluating the AM properties variations due to tissue processing are much more abundant as these parameters can be much better controlled. Tissue processing encapsulates the procedures employed through the AM graft preparation chain, from placenta retrieval, decontamination, AM preparation, and storage and treatment until the moment of transplantation (Aykut et al. 2015; Jirsova and Jones 2017). As the influence of these factors can be rather straightforwardly and rigorously evaluated, numerous studies were devoted to elucidating the effect of AM decontamination/sterilization procedure (Singh et al. 2006; Smeringaiova et al. 2017), graft structure and cellular viability and content modification (intact, or denuded AM) (Koizumi et al. 2000a, b; Hopkinson et al. 2006a, b; Duan-Arnold et al. 2015), the type of preparation and storage (freezing, air-drying, lyophilization) (Dhall et al. 2018; Memmi et al. 2022).

Finally, the effect of these factors on the effectiveness of the AM application is always additionally modulated by the individuality of the treated subject, i.e., by its physiological/pathological conditions, including its sensibility to the AM treatment at the moment of the AM application.

The closure kinetics of chronic wounds usually progresses in two phases; the first is characterized by relatively rapid progress and lasts for the first 5 to 20 weeks of healing with a closure level of more than 50%. The following phase of healing is characterized by a slower progression of wound closure with a less steep curve (Herbin et al. 1993; Venault et al. 2019; Becerra-Bayona et al. 2020). Herein, these two stadia are described as progressive (p-phase) and terminal (e-phase) (Svobodova et al. 2022).

In standard AM preparation for clinical use, several tens of AM sheets are typically prepared from one placenta without keeping exact track of the sub-region origin, except when the targeted region is very specific, e.g., the umbilical part (Cognard et al. 2022). Thus, the intra-placental variations are mostly impossible to survey in clinical applications. However, the track of AMs obtained from individual placentas is rigorous, as required by legislation, and therefore, should the inter-placental differences in AM healing features be prevailing the other factors, they could be potentially detectable by evaluating its healing effect, e.g., by assessing the wound closure rate (DiDomenico et al. 2016; Valiente et al. 2018; Johnson et al. 2021).

In this study, we evaluated the effect of the cryopreserved AMs retrieved from different donors on the efficiency of wound healing (wound closure), intending to understand whether the inter-placental variations could be dominant in wound healing progress or if they are suppressed by the processing/application chain and the individual patient status at the moment of application.

## Materials and methods

The study followed the Ethics Committee’s standards of three participating institutions (1st Medical Faculty of Charles University, General Teaching Hospital, University Hospital Motol, and Na Homolce Hospital, all in Prague) and adhered to the tenets set out in the Declaration of Helsinki.

### AM grafts preparation

After obtaining informed consent from placenta donors, the placenta and blood for serological examination were retrieved. All donors were negative for hepatitis B and C, syphilis, and HIV, C-reactive protein was < 20 mg/l). The serology was repeated after 6 months. The AM grafts were prepared as described earlier (Svobodova et al. 2022). Shortly, all placentas were obtained by elective cesarean section between 38 to 39 (from 38 weeks + 1 day to 39 weeks + 4 days) gestational week in the Motol University Hospital, Prague, from donors with no serious systemic or genetic diseases. Before further processing, placentas were visually inspected for injuries and visible pathologies. Then the tissue (placenta/AM) was decontaminated at room temperature using BASE•128 (Alchimia, Ponte San Nicolò, Italy) for 24 h (± 2 h) at 37 °C. AM sheets were rinsed, stretched on Sanatyl support (Tylex, Letovice, Czech Republic), and cut into desired-sized patches (varying from 2 × 2 cm up to 7 × 11 cm). Finally, AM pieces were placed into Dulbecco’s Modified Eagle Medium (Gibco™ DMEM 32,430,027, Thermo Fisher Scientific) in 50% glycerol (Dr. Kulich Pharma, Czech Republic) and stored at − 80 °C. After six months, tissues with negative microbiology and serology test results were released for grafting**.**

### Patients

The presented study enrolled 16 patients suffering from chronic nonhealing wounds (lasting more than 6 weeks before AM application, range 6 to 1408 weeks, average 139 weeks). Twelve wounds were venous, one arterial, one diabetic origin, one wound was linked to fasciotomy, and one to physical trauma. The inclusion and exclusion criteria have been described previously (Svobodova et al. 2022), shortly, the inclusion criteria were: age ≥ 18 years, the presence of resistant NHW with a duration of more than 6 weeks, and wound extending through the entire thickness of the skin. Exclusion criteria were: tendon or bone exposure in the wound, allergy to antibiotics used for AM decontamination, transcutaneous oximetry value below 30 mmHg for patients with diabetes mellitus, known history of AIDS or HIV, ankle-brachial index (ABI) < 0.5, for all patients except those with diabetes mellitus, suspicious for cancer or history of radiation at the wound site, severe (uncontrolled) systemic disease, or planned surgical intervention in the next six months.

The average age of the patients was 66.8 years (33 to 82), with 4 females and 12 males. Altogether, 22 defects (D) were followed. Before starting the treatment using AM, the wound size varied between 0.99 and 50.51 cm^2^, averaging 12.88 ± 14.54 cm^2^. The wound resistance to treatments before the AM application spanned from 6 to 1408 weeks, averaging 12.88 weeks. The complete healing lasted from 5 to 105 weeks, averaging 31.14 weeks.

### Input data selection and measurement

The efficiency of AM grafts obtained from 9 placentas was analyzed. The mean age of placenta donors was 34 years (26–38). AM sheets from each placenta were distributed to at least three patients, and at least ten AM sheets from individual placentas had to be used to include the placenta in the evaluation. All patients (16 in total) reached complete healing. The data from the p-phase only were evaluated (for the p-phase description, see the introduction and discussion section), which in most cases represents the first 10 to 20 weeks of the treatment. The efficiency score was evaluated as the relative wound closure; the wound size on the day of AM application was used as the baseline (100%), and the percentage of wound area change 7 days after the AM application was evaluated. The size of the wound was assessed as described previously (Svobodova et al. 2022). Briefly, the wound was photo-documented with a scale in the proximity of the wound. The wound size was determined by manually tracing the wound border on calibrated images with an automatic determination of the area using NIS-Elements software (Laboratory Imaging, The Czech Republic). The mean efficiency for each placenta, expressed as an average of wound area reduction (in %), was calculated from at least 10 applications.

### Statistical analysis

First, the data sets for individual placentas were checked for the normality by Saphiro-Wilk’s test. The results showed that not all sets were of normal distribution, so the Kruskal–Wallis test was applied to check if a statistically significant difference could be detected. Finally, Dunn’s test for performing multiple pairwise-comparison between the means of individual placentas was used. All the evaluations were performed using the R and RStudio package (RStudio 2020).

## Results

**Fig. 1:**
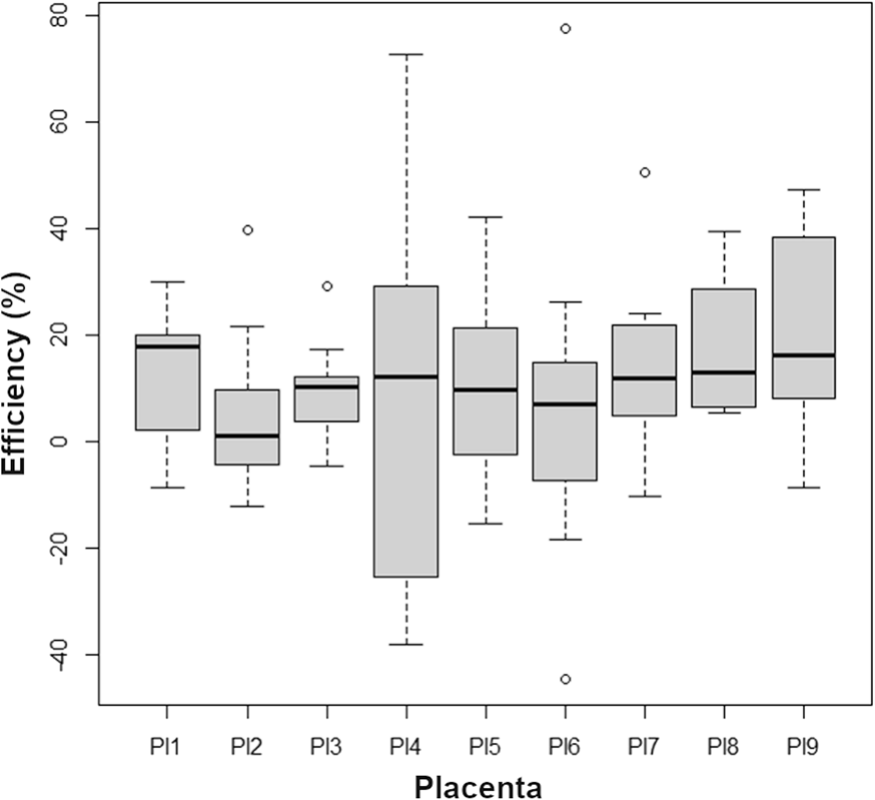
A statistical representation of the efficiencies of AMs originating from different placentas

The results represent the analysis of AM efficiency on non-healing wound treatment over 5 years (2017–2022). The treatment of the “non-healing” wounds using AMs included in the study led to a complete wound closure at the end of the treatment. For the evaluation of the AM efficiency, only the p-phase of the healing progress was used (for details, see the Discussion section). Our evaluation of the AM efficiency shows that the 7-day average wound closure (% of wound surface) in the p-phase of healing after the application of cryopreserved AMs varied from 5.7 to 20.99% (medians from 1.07 to 17.75). The values for individual placentas are summarized in Table 1**,** and statistics are visualized in Fig. 1. These results suggest that the average wound closure rate when using cryopreserved AM for non-healing wounds is 12.17 ± 20.12% (average ± standard deviation) of the wound surface 7 days after AM application.

**Table 1:** The average healing efficiency of the placentas is expressed as wound area reduction in % seven days after AM application

	Average ± SD	Median	Saphiro-Wilk test *p* value
Placenta 1	13.67 ± 12.98	17.75	0.19
Placenta 2	5.70 ± 14.47	1.07	0.18
Placenta 3	10.13 ± 8.66	10.24	0.63
Placenta 4	6.83 ± 32.75	12.29	0.60
Placenta 5	12.13 ± 17.65	9.65	0.77
Placenta 6	7.26 ± 29.11	7.02	0.33
Placenta 7	13.46 ± 14.13	11.87	0.33
Placenta 8	18.06 ± 13.07	12.87	0.01
Placenta 9	20.99 ± 17.19	16.21	0.50

The records for individual defects treated by each placenta evaluated in this study are summarized in Table 2. The negative values represent a temporary worsening of the wound against the baseline. While the spread of the values measured after AM application was rather important for individual placentas (e.g., for placenta 4 ranging from − 38.20 to 72.73% of wound area closure one week after application, see Table 2), resulting in high values of standard deviations, the values of both mean and median were relatively coherent, ranging from 5.70 to 20.99 and 1.07 to 17.75 respectively.

**Table 2:** Effects of applications of AMs from selected placentas on individual defects seven days after application time

Placenta 1			Placenta 2			Placenta 3		
Change (%)	Defect	Application date	Change (%)	Defect	Application date	Change (%)	Defect	Application date
30.00	P1-D1	2017–10-05	− 1.32	P4-D1	2019–02-26	17.36	P6-D1	2018–07-31
14.01	P2-D1	2018–06-26	− 12.14	P4-D2	2019–02-26	11.38	P6-D1	2018–08-07
− 6.44	P3-D2	2018–07-10	− 6.25	P5-D1	2019–01-15	12.08	P2-D1	2018–07-10
30.00	P1-D1	2017–10-23	9.70	P6-D4	2019–01-02	29.23	P3-D2	2018–07-17
20.00	P1-D1	2017–10-28	− 1.25	P6-D4	2019–01-15	3.76	P3-D1	2018–07-17
20.00	P1-D1	2017–11-01	− 4.29	P5-D1	2019–01-02	11.80	P3-D1	2018–07-03
19.66	P3-D2	2018–07-24	39.66	P4-D2	2019–04-02	−4.72	P3-D2	2018–07-03
15.85	P3-D1	2018–08-07	21.71	P7-D1	2019–04-24	8.99	P5-D1	2018–09-11
2.24	P3-D2	2018–08-07	3.39	P8-D1	2019–04-24	9.09	P3-D2	2018–09-04
-8.60	P2-D1	2018–07-03	7.81	P8-D1	2019–04-17	2.30	P3-D1	2018–09-04

Placenta 4			Placenta 5			Placenta 6		
Change (%)	Defect	Application date	Change (%)	Defect	Application date	Change (%)	Defect	Application date
− 34.62	P5-D2	2018-10-16	8.73	P6-D4	2018-11-27	− 18.52	P8-D1	2019-04-10
− 8.48	P5-D1	2018-10-16	18.47	P5-D1	2018-12-11	− 13.60	P9-D1	2019-07-02
− 29.47	P5-D2	2018-10-02	4.07	P6-D4	2018-11-06	3.29	P9-D1	2019-07-09
− 38.20	P3-D1	2018-10-02	−2.45	P4-D1	2019-02-12	− 44.52	P9-D1	2019-06-11
19.37	P5-D2	2018-09-18	10.57	P6-D4	2018-11-13	− 0.90	P9-D1	2019-04-09
5.22	P3-D1	2018-10-16	−15.56	P5-D1	2018-11-13	7.02	P9-D1	2019-04-30
38.57	P6-D1	2018-09-25	42.25	P3-D1	2018-11-20	15.52	P8-D1	2019-04-30
19.77	P4-D1	2019-03-05	−5.17	P6-D4	2018-11-20	26.28	P7-D1	2019-04-30
21.66	P4-D2	2019-03-05	39.07	P5-D2	2018-10-30	14.04	P8-D1	2019-05-02
− 21.27	P5-D1	2018-10-02	21.34	P5-D1	2018-10-30	77.55	P8-D1	2019-05-07
36.66	P6-D1	2018-10-09				13.71	P4-D1	2019-06-25
72.73	P6-D2	2018-10-09						

Placenta 7			Placenta 8			Placenta 9		
Change (%)	Defect	Application date	Change (%)	Defect	Application date	Change (%)	Defect	Application date
10.21	P10-D1	2021-03-01	5.51	P14-D1	2022-03-15	16.28	P16-D1	2022-02-22
21.88	P11-D1	2021-03-22	36.50	P15-D1	2019-09-10	32.64	P16-D2	2022-02-22
14.41	P12-D1	2020-08-04	16.47	P14-D1	2022-03-01	4.38	P13-D1	2021-12-21
4.35	P11-D1	2021-04-12	9.66	P14-D1	2022-01-25	−8.61	P13-D1	2022-01-10
50.55	P12-D1	2020-10-27	5.89	P9-D1	2019-08-13	40.25	P16-D2	2022-02-15
23.55	P12-D1	2020-08-11	5.87	P4-D1	2019-07-23	0.67	P13-D1	2022-02-14
− 10.34	P11-D1	2021-03-17	12.87	P15-D1	2019-07-24	14.08	P13-D2	2021-12-13
24.06	P10-D1	2021-03-17	20.93	P15-D1	2019-07-29	11.99	P14-D1	2022-05-31
4.90	P11-D1	2021-03-01	7.31	P15-D1	2019-08-27	38.93	P16-D1	2022-02-01
− 4.11	P11-D1	2021-02-12	38.17	P9-D1	2019-08-27	37.73	P16-D2	2022-02-08
13.54	P10-D1	2021-04-12	39.44	P15-D1	2019-09-25	47.42	P16-D2	2022-01-03
19.15	P10-D1	2021-02-08				16.14	P14-D1	2022-05-17
9.64	P10-D1	2021-04-19						
6.67	P13-D1	2022-02-28						

*P* designates the subject number, *D* is the defect number of the given subject

The results of Saphiro-Wilk’s test revealed dispersed values for the normality of individual sets. Therefore, Levene’s test was performed to decide whether parametric analysis (ANOVA) could be used. The resulting p-value of 0.008 indicated the data’s non-normal character; thus, the Kruskal–Wallis test was applied. Its result showed no statistically significant difference (*p* = 0.492) between individual placentas. Therefore, it is legitimate to conclude that, in general, there is no important difference in the efficacy of AMs originating from different placentas on wound healing.

## Discussion

In this study, we aimed to determine whether some significant differences in the healing efficiency of AM applied to non-healing wounds can be traced between AM sheets prepared from different placentas and to establish mean wound closure. As mentioned in the introduction, the existing studies are somewhat controversial concerning the inter- and intra-placental variation in the presence/concentration of wound healing factors (Avila-Gonzalez et al. 2015; Centurione et al. 2018). However, if such differences can be detected in the AM clinical applications for wound healing, it would help to orient more targeted studies on how to evaluate the placentas healing potential (both in inter-placental and intra-placental respect) and perhaps avoid the use of less efficient placentas for their application, which could spare an important amount of preparative time and shorten the healing period.

Herein, we defined the placenta inclusion parameters at three different levels. First, the patient's positive reactive response to the AM application healing procedure was critical. Therefore, only patients with good healing progress and complete final healing were included. Second, only the first progressive phase of healing was included in the evaluation. As we reported previously, the healing progress of well-reacting patients in our clinical study could be fitted with asymptotic function (Svobodova et al. 2022). This is characterized by rather rapid progress in the initial phases, which is then progressively slowed down in the final healing period when the last few percent of the closure generally heal much slower than at the healing onset (Becerra-Bayona et al. 2020).

Moreover, the inaccuracies in the wound size determination increase with the smaller wound size and with the absolute differences in the wound size between the measurements. Therefore, our interest was to utilize the period when the wound size and its changes were the most important, typically in the first 10–20 weeks of healing. Furthermore, the patients with multiple wounds were preferred as this would allow us to, at least partly, evaluate the subject/defect status factor.

The rate of wound healing (% of wound closure per week) in the p-phase of our patients is consistent with the regularly observed one (Bull et al. 2022). Our results suggest that more than individual placentas' properties, patients' physiological status predominantly influences the wound closure progress. We suppose that important variation of obtained values found almost for each placenta (with one exception—Pl8) reflects the patient’s or wound's immediate physiological/pathological condition. This is supported by the observation that the average healing efficiency of all 9 analyzed placentas did not statistically differ. However, it is necessary to consider the limitations of this study, such as the small number of placentas (9) and the variability in patients' parameters and their wounds.

The results show that although the average values of placenta efficiency may differ quite notably (more than double the value between Pl1 and Pl6), the analysis does not confirm the statistical significance. We have also observed case-by-case differences in reaction to the AM application. In some cases, we recorded different responses of two wounds of the same subject being treated by the AM from the same placenta. E.g., when treated with AMs from placenta 1 (Pl1), the reaction of the defects P3-D1 and P3-D2, which are the two defects of the same subject, was quite different even though they were treated on the same visit day (2018-08-07), closure 15.85 vs. 2.24%, respectively. A similar situation could be observed for the P5-D1 and P5-D2 (again two defects of the same patient) wherein two weeks, the defect P5-D1 changed its reaction from positive 21.34% (2018-10-30) to negative − 15.56% (2018-11-13) when treated by AMs from placenta 5 (Pl5). At the same time, the reaction of P5-D2 was twice as important as that of P5-D1 on the same application date (2018-10-30, 39.07% vs. 21.34%, respectively). Another example of reaction variation on the AM application can be detected for the defect P9-D1, which had a very different response on the AMs from placenta 6 in two months (2019-04-30 vs. 2019-06-11). All the data suggest that if there are intra- and inter-placental differences, they are overridden mainly by the actual health status of the subject or even the status of its individual wounds (due to the microbial, blood circulation, or other possible conditions, which may affect the healing process).
